# Only 32.3% of Breast Cancer Families with Pathogenic Variants in Cancer Genes Utilized Cascade Genetic Testing

**DOI:** 10.3390/cancers15215218

**Published:** 2023-10-30

**Authors:** Konstantinos Agiannitopoulos, Kevisa Potska, Anastasia Katseli, Christina Ntogka, Georgios N. Tsaousis, Georgia Pepe, Dimitra Bouzarelou, Nikolaos Tsoulos, Athanasios Papathanasiou, Dimitrios Ziogas, Vassileios Venizelos, Christos Markopoulos, Rodoniki Iosifidou, Sofia Karageorgopoulou, Stylianos Giassas, Ioannis Natsiopoulos, Konstantinos Papazisis, Maria Vasilaki-Antonatou, Amanta Psyrri, Anna Koumarianou, Dimitrios Matthaios, Eleni Zairi, Alexandru Blidaru, Eugeniu Banu, Dan Corneliu Jinga, Şahin Laçin, Mustafa Özdoğan, Eirini Papadopoulou, George Nasioulas

**Affiliations:** 1 Genekor Medical S.A., 15344 Athens, Greece; v.potska@genekor.com (K.P.); n.katseli@genekor.com (A.K.); c.dogka@genekor.com (C.N.); gtsaousis@genekor.com (G.N.T.); gpepe@genekor.com (G.P.); d.bouzarelou@genekor.com (D.B.); ntsoulos@genekor.com (N.T.); a.papathanasiou@genekor.com (A.P.); eirinipapad@genekor.com (E.P.); gnasioulas@genekor.com (G.N.); 2 General Hospital of Athens “LAIKO”, 11527 Athens, Greece; ziogasdc@gmail.com; 3 Metropolitan Hospital, 18547 Athens, Greece; bennievenizelos@gmail.com; 4 Athens Medical Center, 15125 Athens, Greece; cmbreast@gmail.com; 5 Theagenio Anticancer Hospital, 54639 Thessaloniki, Greece; rodoniki69@gmail.com; 6 IASO, General Maternity and Gynecology Clinic, 15123 Athens, Greece; skarageorgopoulou@iaso.gr (S.K.); sgiassas@yahoo.com (S.G.); 7 Interbalkan Medical Center of Thessaloniki, 55535 Thessaloniki, Greece; ynatsiopoulos@yahoo.gr; 8 Euromedica General Clinic, 54636 Thessaloniki, Greece; k.papazisis@oncomedicare.com; 9 Metropolitan General Hospital, 15562 Athens, Greece; vasilakimaria@gmail.com; 10 Section of Medical Oncology, Attikon University Hospital, National and Kapodistrian University of Athens, 12462 Athens, Greece; psyrri237@yahoo.com (A.P.); akoumari@yahoo.com (A.K.); 11 General Hospital of Rhodes, 85133 Rhodes, Greece; dimalexpoli@yahoo.com; 12 St. Luke’s Hospital, 55236 Thessaloniki, Greece; zairi.eleni@gmail.com; 13 Alexandru Trestioreanu Bucharest Oncology Institute, 022328 Bucharest, Romania; alexandrublidaru@yahoo.com; 14 Saint Constantin Hospital, 500299 Brasov, Romania; eugeniu.banu@spitalulsfconstantin.ro; 15 Neolife Medical Center, 013812 Bucharest, Romania; danjinga2002@yahoo.com; 16 Department of Medical Oncology, Koc University Faculty of Medicine, 34010 Istanbul, Turkey; sahin.lacin@hotmail.com; 17 Division of Medical Oncology, Memorial Antalya Hospital, 07025 Antalya, Turkey; ozdoganmd@yahoo.com

**Keywords:** cascade family testing, hereditary breast cancer, next-generation sequencing

## Abstract

**Simple Summary:**

The aim of this study was to explore the utility of cascade family testing (CFT) in families of breast cancer patients who carry specific genetic variations. We conducted genetic analysis in a group of breast cancer patients and identified genetic variations in over 20% of them; thus, CFT was recommended to their first-degree relatives. Among those tested, the majority were asymptomatic females and mainly offspring, siblings, or parents of the patients. The study showed that CFT was most commonly performed in families with high-risk genetic findings, and we concluded that CFT could be a valuable approach for preventing hereditary cancers by identifying at-risk family members. We also emphasized the need for improved genetic counseling and communication to ensure effective implementation.

**Abstract:**

Background: Hereditary cancer predisposition syndromes are responsible for approximately 5–10% of all diagnosed cancer cases. In order to identify individuals at risk in a cost-efficient manner, family members of individuals carrying pathogenic alterations are tested only for the specific variant that was identified in their carrier relative. The purpose of this study was to investigate the clinical use and implementation of cascade family testing (CFT) in families of breast cancer patients with pathogenic/likely pathogenic variants (PVs/LPVs) in cancer-related predisposition genes. Methods: Germline sequencing was carried out with NGS technology using a 52-gene panel, and cascade testing was performed by Sanger sequencing or MLPA. Results: In a cohort of 1785 breast cancer patients (families), 20.3% were found to have PVs/LPVs. Specifically, 52.2%, 25.1%, and 22.7% of patients had positive findings in high-, intermediate-, and low-penetrance breast cancer susceptibility genes, respectively. Although CFT was recommended to all families, only 117 families (32.3%) agreed to proceed with genetic testing. Among the first-degree relatives who underwent CFT, 70.3% were female, and 108 of 121 (89.3%) were cancer free. Additionally, 42.7%, 36.7%, and 20.6% were offspring, siblings, and parents of the subject, respectively. Our data suggest that CFT was mostly undertaken (104/117, 88.8%) in families with positive findings in high-risk genes. Conclusions: Cascade family testing can be a powerful tool for primary cancer prevention by identifying at-risk family members. It is of utmost importance to implement genetic counseling approaches leading to increased awareness and communication of genetic testing results.

## 1. Introduction

Hereditary breast cancer refers to the subgroup of breast cancer cases with inherited genetic susceptibility due to specific genetic pathogenic or likely pathogenic variants (PVs/LPVs) showing familial clustering. Although the vast majority of breast cancers arise from acquired DNA damage primarily shaped by external environmental and lifestyle elements, hereditary breast cancer diagnoses are caused by PVs/LPVs in genes that are critical for upholding the stability of DNA within cells [1].

Genes have been categorized into different groups based on their association with the risk of developing breast cancer [2]. High-risk genes are those that, when mutated, significantly increase the risk of cancer development, with a risk greater than four times (>4×) that of the general population over a lifetime. Moderate-risk genes, on the other hand, confer a risk of two to four times (2–4×) compared to the general population. Low-risk genes are associated with a less than two times (<2×) increased risk of cancer. For some genes, there is limited or insufficient evidence available regarding their link to cancer and the extent of the associated risk [3]. It is important to note that this classification is dynamic and continuously updated based on clinical evidence from various sources, with the aim of achieving a consensus within the scientific community.

*BRCA1* and *BRCA2* (known as BReast CAncer genes 1 and 2) are the most widely recognized breast cancer susceptibility genes. PVs/LPVs in these genes markedly increase the risk of developing breast and/or ovarian cancer. These PVs/LPVs adhere to an autosomal dominant inheritance pattern, substantially increasing the risk of cancer development in first-degree relatives inheriting them compared to the general population [4]. In addition to the *BRCA1* and *BRCA2* genes, there are several other genes associated with an increased risk of breast cancer. Some of these genes include *TP53*, *CHEK2*, *ATM*, *PALB2*, *PTEN*, *STK11*, *CDH1*, *NF1*, *RAD51C*, and *RAD51D* [5].

Individuals with a family history of breast cancer, particularly a history characterized by early-age diagnoses and multiple primary cancers, may opt for genetic testing to identify PVs/LPVs occurring in high-risk genes. Detection of such a variant could trigger cascade family testing (CFT), a process involving the genetic testing of higher-risk family members to determine their genetic status. CFT serves to identify individuals with genetic PVs/LPVs before symptoms manifest [6]. This early identification empowers healthcare providers to initiate surveillance measures and preventive strategies, thereby facilitating early intervention and potentially improved outcomes. The practice of cascade testing facilitates personalized risk evaluation and counseling by recognizing individuals at heightened susceptibility to hereditary cancer. Individuals and their families are empowered to make informed choices regarding cancer screening, preemptive surgeries, and lifestyle modifications to enhance overall well-being [7]. CFT for genes with moderate or low penetrance, which do not have clear-cut management recommendations, is a crucial query. This is further complicated by the general ambiguity surrounding risk interpretation for many genes with moderate penetrance. The lack of consensus on the best clinical approach for pathogenic variants (PVs) in these genes underscores the necessity for specialized genetic counseling education [8].

CFT is a crucial and fundamental element in the field of genetic counseling and testing approaches, playing a significant role in uncovering the innate factors contributing to susceptibility to breast cancer among family members. This approach holds substantial value in identifying these factors. It goes beyond the initial individual, typically referred to as the proband, who has received a diagnosis of a breast cancer-related PV/LPV [9]. One of the primary challenges is the complexity of acquiring comprehensive and well-informed consent from family members for genetic testing. This procedure is rife with ethical, emotional, and psychological intricacies, spanning from concerns about privacy to addressing the consequences of test results. The likelihood of a chain reaction of emotional impact on individuals who become informed about their genetic predisposition should not be underestimated, emphasizing the necessity of robust psychological support mechanisms [10].

The aim of this study was to investigate the clinical use and implementation of CFT within families of breast cancer patients carrying PVs/LPVs.

## 2. Materials and Methods

We conducted a thorough retrospective investigation involving a total of 1785 breast cancer patients belonging to 1785 families, who were specifically referred to our laboratory for the purpose of undergoing genetic testing. This research was conducted within a private diagnostic laboratory, where patients were not chosen based on rigid criteria for genetic testing. Instead, every individual received genetic counseling including explanations about the importance of molecular testing by a physician and/or geneticist from our laboratory. The patients shared details about their personal and familial backgrounds and gave their consent before undergoing molecular genetic testing. They also gave permission for their data to be used anonymously for research or scientific publications.

As previously described [11], our focus encompassed a comprehensive panel of 52 genes that are known to be associated with a predisposition to hereditary cancers (Table S1). Among these genes were the following breast cancer-associated genes: *ATM*, *BARD1*, *BLM*, *BRCA1*, *BRCA2*, *BRIP1*, *CDH1*, *CHEK2*, *NBN*, *NF1*, *PALB2*, *PTEN*, *RAD50*, *RAD51C*, *RAD51D*, *STK11*, and *TP53*. These genes were selected for cancer predisposition testing based on extensive scientific research and established guidelines (Table S2). 

The analysis of these 52 genes was carried out utilizing a highly effective and targeted approach known as a capture-based method, which was seamlessly integrated with cutting-edge next-generation sequencing (NGS) technology. This advanced methodology was generated through the application of the DNBSEQ-G400 technology. In the course of the sequencing procedure, a fundamental step involved aligning the genetic reads to a curated reference sequence referred to as GRCh37. This alignment was essential for ensuring accuracy and precision in the identification of any deviations or alterations within the genetic sequences. Importantly, any identified sequence variations were meticulously evaluated in the context of a specific transcript that holds clinical significance. 

One particularly noteworthy aspect of our approach was the incorporation of the capture-based method. This method enables the assessment of copy number variations (CNVs), thus enriching our understanding of the genetic landscape related to hereditary cancer predisposition.

**Figure 1:**
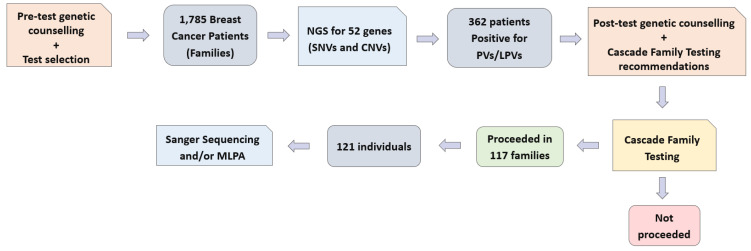
Schematic representation of the workflow used in this study.

Furthermore, we executed a comprehensive cascade testing protocol involving 121 individuals from 117 families. This process involved either Sanger sequencing or the utilization of multiplex ligation-dependent probe amplification (MLPA) techniques. These advanced methodologies enabled us to scrutinize specific genetic regions of interest, thereby ensuring a rigorous assessment of potential genetic pathogenic variants. A clear overview of the workflow is provided as a visual representation below (Figure 1).

The *p*-values were calculated utilizing Fisher’s exact test. A *p*-value < 0.05 was considered to be statistically significant.

The study was conducted in accordance with the Declaration of Helsinki and approved by the Ethics Committee of the Hellenic Breast Surgeons Society (1 September 2023).

## 3. Results

Our comprehensive study of a cohort of 1785 individuals who had been diagnosed with breast cancer provides novel insight into the genetic landscape of the disease. Among this group, 362 patients, accounting for approximately 20.3% of the total cohort, carried a significant genetic distinction—the presence of either pathogenic variants (PVs) or likely pathogenic variants (LPVs). These variants represent crucial genetic alterations that play a substantial role in the development and progression of breast cancer. By delving deeper into the specifics of these genetic alterations, we found that the positively identified patients could be categorized into distinct groups based on the nature of the variations within their genomes.

**Figure 2:**
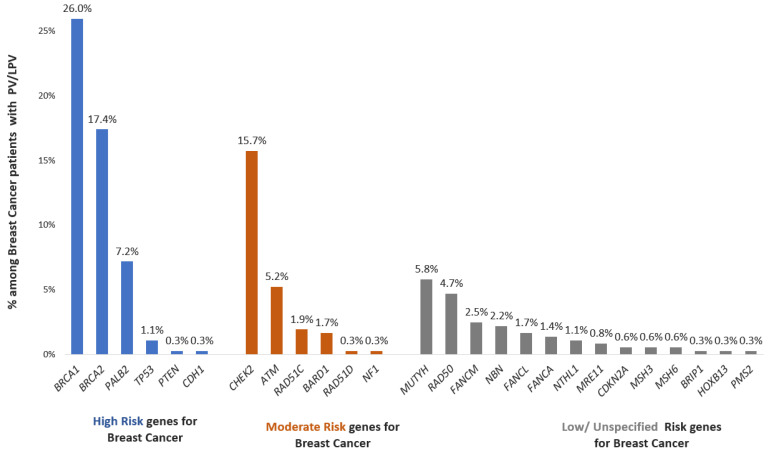
Percentages of genes with P/LP variants among breast cancer patients with positive findings. Genes are divided into different categories according to the associated risk for breast cancer.

A noteworthy 52.2% of these individuals exhibited variations within high-penetrance cancer susceptibility genes. Further analysis revealed that 25.1% of the patients carried variations in moderate-penetrance cancer susceptibility genes. Intriguingly, 22.7% of the patients were identified with a pathogenic/likely pathogenic variant in low/unspecified-penetrance cancer susceptibility genes (Figure 2). 

Despite the strong and widely acknowledged recommendation for the integration of genetic counseling and CFT within all families affected by potential genetic conditions, the actual uptake of this vital process was notably low. Among the targeted group of 362 families, only 117 families, comprising approximately 32.3% of the total, chose to move forward with the genetic testing procedure. In these 362 families with potentially significant genetic variants, we estimated that about 1246 first-degree relatives could benefit from CFT; however, 121 individuals, or 9.7%, pursued CFT. Even within the same family, not all members chose to undergo genetic testing.

The average age of the patients who initiated the testing was 46 years, and the median age was 45 (26–80). In parallel, the average age of the first-degree relatives who actively engaged in the genetic testing process was noted to be 40 years (Table 1). This age difference between the probands and their tested relatives yielded a statistically significant result (*p* < 0.0001), signifying a noteworthy discrepancy. 

Among the individuals who opted for genetic counseling and CFT within the group of first-degree relatives, a substantial majority, approximately 70%, were female (Table 1). 

Of the total 121 individuals who engaged in the genetic testing process, a significant proportion, specifically 108 individuals, accounting for 89.3% of the tested relatives, displayed no discernible symptoms or indications related to cancer (Table 1). 

**Figure 3:**
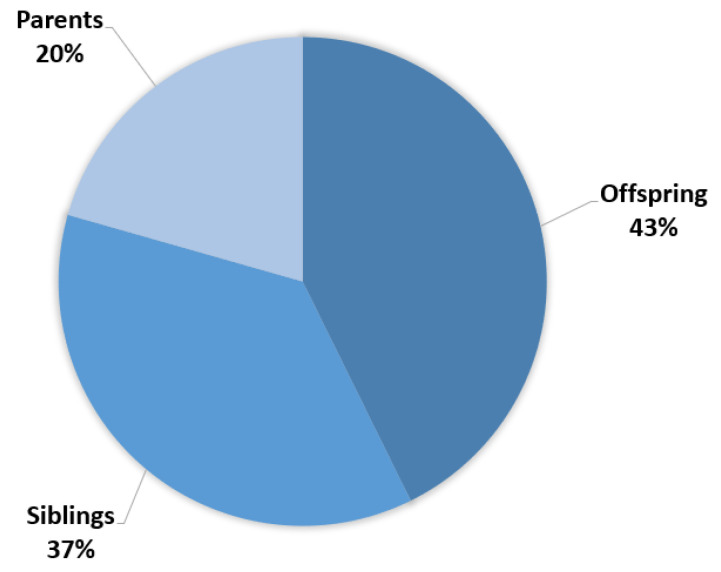
Percentage of first-degree relative categories undergoing cascade family testing.

The median duration of the cascade testing process, an essential aspect of genetic testing that involves tracing the genetic variants through a family tree, was calculated to be nine months. Within the group of 121 individuals who pursued genetic testing, a detailed stratification of their relationships to the patients is shown in Figure 3. Approximately 42.7% of the tested individuals were offspring, indicating a concern for future generations and the desire to understand potential risks that could affect their children. About 36.7% were siblings, reflecting the pivotal role of siblings in supporting and understanding familial health histories. Finally, 20.6% were parents of the probands, illustrating the multi-generational impact of the genetic condition and the parental commitment to obtaining crucial information for their own well-being as well as that of their offspring.

Out of the total instances of CFT among the 117 families, a significant 88.8% (104 families) were conducted within high-risk families.

## 4. Discussion

Approximately 5–10% of all breast cancers are attributed to the syndrome known as hereditary breast and ovarian cancer (HBOC) [12]. The identification of pathogenic and likely pathogenic variants (20.3%) in a significant proportion of the current cohort underscores the role of genetics in disease manifestation. The diverse distribution of these variants across high- (52.5%), moderate- (25.1%), and low-penetrance (22.7%) genes accentuates the multifactorial nature of breast cancer susceptibility. This insight not only advances our understanding of the genetic mechanisms driving breast cancer but also lays a foundation for more targeted and personalized approaches to diagnosis, treatment, and risk assessment of individuals affected by this complex disease [13].

The observation that only a fraction of families (32.3%) followed through with recommended genetic counseling and testing despite its acknowledged importance emphasizes the complex interplay of factors shaping individuals’ choices in healthcare [14]. Our results agree with a growing number of studies that have examined the uptake of cascade family testing for hereditary cancer and show generally that this is lower than 30% among the eligible first-degree relatives [15,16]. Another important issue is that after a careful inspection of the 362 families with a proband identified as carrying a P/LP variant, we computed that approximately 1246 first-degree relatives would be eligible for CFT, but only a small number of individuals (121 individuals, 9.7%) proceeded with testing even in families that responded to CFT. Therefore, more efforts should focus on encouraging first-degree relatives in these families to pursue testing and reducing the cost of CFT approaches.

An interesting result of our study is the gap between the age average of the probands up taking the genetic testing and that of their first-degree relatives at the time of CFT. This discrepancy might reflect the diverse motivations and concerns that individuals at different life stages hold regarding genetic testing. Younger individuals, often less burdened by health issues or more distant in time from family members’ diagnoses, could perceive genetic testing as a preemptive measure to shape their future health decisions. The significant age gap between the probands and their tested relatives not only underscores the impact of age on the decision to pursue genetic testing but also points to the potential influence of other factors such as family dynamics, health histories, and personal beliefs. The decision to opt for genetic testing is multifaceted and influenced by numerous considerations, ranging from personal health awareness to familial responsibilities [5]. Our findings collectively highlight the need for a comprehensive understanding of the diverse motivations and barriers that individuals face when considering genetic testing, ultimately guiding efforts to enhance the accessibility and acceptance of this critical component of personalized healthcare [17].

Furthermore, the comprehensive demographic stratification of tested individuals underscores the complexity of familial dynamics and the diverse motivations for undergoing genetic testing. The proactive involvement of individuals who displayed no symptoms (89.7%) underscores the increasing awareness of the potential benefits of preemptive health management. Additionally, the distribution of tested individuals across different relationship categories emphasizes the collaborative and interconnected nature of family health. The statistical data regarding the sex distribution, absence of cancer symptoms, testing duration, and relationship categories provides a rich context for understanding the choices and motivations behind the decision of first-degree relatives to pursue genetic testing. These insights collectively contribute to a deeper comprehension of the multifaceted considerations that drive individuals to engage in genetic counseling and testing, ultimately shaping more informed healthcare decisions and potentially enabling the implementation of effective preventive strategies [18]. 

Factors associated with increased uptake of CFT are higher educational level and socioeconomic factors, effective communication with relatives, and comfort with disclosing the results at the proband level, while at the family-level, female sex has an additive effect [19], a conclusion also revealed by our study. This highlights the critical role that women often play in health-related decisions within families, underscoring their active engagement in seeking valuable genetic insights to enhance their understanding of potential health risks and probably also reflects the strong belief among patients and their families that breast cancer-related genetic findings at least will not affect male relatives. However, breast cancer genes are also associated with increased risk for other cancers affecting both sexes.

The cumulative data emphasize the significance of both identifying shared genetic risk within families and focusing on high-risk gene families during the testing process. The prevalence of shared PVs/LPVs within tested first-degree relatives highlights the tangible impact of genetics on familial health patterns, while the strategic prioritization of high-risk gene families showcases a targeted approach to risk assessment and prevention [20]. The application of CFT in families with probands carrying genes with moderate or low penetrance, particularly those without established management protocols, is a critical area of inquiry. This is further complicated by the widespread uncertainty surrounding how to assess the risk of many genes with moderate penetrance [7]. The lack of consensus on the most effective clinical approach for addressing PVs in these genes emphasizes the pressing need for specialized genetic counseling education. This education is essential for providing tailored guidance on risk management for individuals carrying these variants, ensuring they receive the most informed and personalized care possible. As such, advancing genetic counseling education becomes a pivotal step towards improving clinical care and support for those with PVs in moderate- and low-penetrance genes [8].

**Figure 4:**
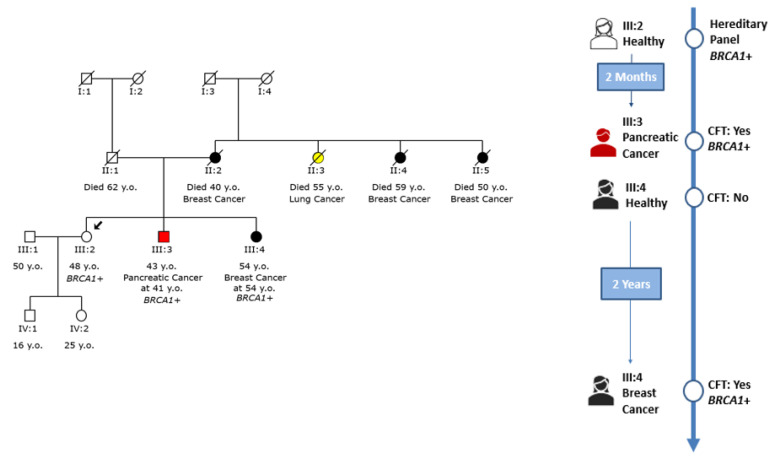
An example of cascade testing within a family with a *BRCA1* pathogenic variant.

An example of cascade testing within a family with a *BRCA1* pathogenic variant is shown in Figure 4. In this particular family, the initial individual of interest, marked as III:2 within the family tree, underwent genetic testing due to the family’s notable history of breast cancer and was found to carry a pathogenic variant within the *BRCA1* gene. Following the disclosure of the testing results, the proband’s brother (III:3), who had been diagnosed with pancreatic cancer, was subsequently tested and identified to carry the same variant [21]. 

In contrast, the proband’s sister (III:4) was healthy at the time of her brother’s positive genetic testing outcome. Two years later, her circumstances changed dramatically as she was diagnosed with breast cancer. Considering her diagnosis, she opted to undergo genetic testing that revealed that she also carried the familial variant in the *BRCA1* gene. This finding further underscored the impact of such testing in unraveling familial predispositions to certain cancers and how these insights can potentially influence healthcare decisions, risk assessment, and preventive measures [22]. The journey from the initial proband to the subsequent testing of family members demonstrates the complexity of genetic influences and their profound implications for health outcomes within a family context [23].

A recent study with a large sample size investigated the rate of unexpected pathogenic/likely pathogenic variants in relatives undergoing multigene panel testing (MGPT) instead of the limiting cascade testing for familial PVs/LPVs recommended in the guidelines. In 6.2% of the relatives, an unexpected PV/LPV in a cancer predisposition gene was detected, and in nearly half (2.7% of the relatives that were tested), the unexpected PV/LPV was in a high- or moderate-penetrance gene, offering a change in clinical management in a high proportion of them [24]. A future perspective of our study could include MGPT in relatives to reveal such unexpected findings and empower the value of such an approach.

Certainly, this research, while valuable, is not without limitations. One notable constraint is the relatively small sample size of individuals who underwent CFT. This may potentially influence the generalization of the findings to larger populations. Additionally, a crucial aspect that warrants attention is the absence of follow-up data for both the initial proband and their relatives who underwent testing. This lack of longitudinal information limits our ability to track the progression of or any changes in their genetic status over time, which is essential for a comprehensive understanding of the outcomes. Another noteworthy limitation pertains to the uncertainty surrounding whether some relatives may choose to undergo testing at a different laboratory in the future. This introduces a potential source of variability in the testing process and outcomes, as different laboratories may employ slightly different methodologies or have distinct interpretations of genetic data. This information gap could impact the overall consistency and reliability of the results and should be taken into consideration when interpreting the findings.

Considering these limitations, it is important to acknowledge that while this research provides valuable insights, further studies with larger sample sizes and robust long-term follow-up protocols are needed to strengthen the reliability and applicability of the findings. Additionally, future investigations may benefit from tracking whether relatives pursue testing at alternate laboratories, allowing for a more comprehensive understanding of the genetic landscape within the family context.

## 5. Conclusions

In conclusion, the outcomes of this study highlight the interaction between genetic heritage and the evaluation of risk within families. The significant presence of PVs/LPVs among immediate relatives accentuates the genetic basis of the condition. Moreover, the increased focus on families with high-risk genes during testing exemplifies a well-informed and calculated strategy for risk control. These findings add to the expanding knowledge base regarding genetic testing and its consequences, enhancing our grasp on anticipating, comprehending, and potentially reducing the risk of hereditary disorders within familial contexts.

## Figures and Tables

**Table 1:** Information on individuals who underwent CFT.

Categories	n = 121
Female, n (%)	85 (70.3%)
Male, n (%)	36 (29.7%)
Mean Age, years	40
Asymptomatic, n (%)	108 (89.3%)
Symptomatic, n (%)	13 (10.7%)

## Data Availability

The data presented in this study are available on request from the corresponding author.

## Supplementary Materials

The following supporting information can be downloaded at: https://www.mdpi.com/article/10.3390/cancers15215218/s1, Table S1: 52-genes included in this study; Table S2: Breast cancer associated genes and risk category.

## References

[1] Garutti M., Foffano L., Mazzeo R., Michelotti A., Da Ros L., Viel A., Miolo G., Zambelli A., Puglisi F. (2023). Hereditary Cancer Syndromes: A Comprehensive Review with a Visual Tool. Genes.

[2] Economopoulou P., Dimitriadis G., Psyrri A. (2015). Beyond BRCA: New hereditary breast cancer susceptibility genes. Cancer Treat. Rev..

[3] Sokolova A., Johnstone K.J., McCart Reed A.E., Simpson P.T., Lakhani S.R. (2023). Hereditary breast cancer: Syndromes, tumour pathology and molecular testing. Histopathology.

[4] Lee A., Moon B.I., Kim T.H. (2020). BRCA1/ BRCA2 Pathogenic Variant Breast Cancer: Treatment and Prevention Strategies. Ann. Lab. Med..

[5] Daly M.B., Pilarski R., Yurgelun M.B., Berry M.P., Buys S.S., Dickson P., Domchek S.M., Elkhanany A., Susan Friedman S., Garber J.E. (2020). NCCN Guidelines Insights: Genetic/Familial High-Risk Assessment: Breast, Ovarian, and Pancreatic, Version 1.2020. J. Natl. Compr. Canc. Netw..

[6] Silver E.L., Niell-Swiller M. (2022). Should all patients undergoing genetic testing for hereditary breast cancer syndromes be offered a multigene panel?. Curr. Opin. Obstet. Gynecol..

[7] Frey M.K., Ahsan M.D., Bergeron H., Lin J., Li X., Fowlkes R.K., Narayan P., Nitecki R., Rauh-Hain J.A., Moss H.A. (2022). Cascade Testing for Hereditary Cancer Syndromes: Should We Move Toward Direct Relative Contact? A Systematic Review and Meta-Analysis. J. Clin. Oncol..

[8] Caswell-Jin J.L., Zimmer A.D., Stedden W., Kingham K.E., Zhou A.Y., Kurian A.W. (2019). Cascade Genetic Testing of Relatives for Hereditary Cancer Risk: Results of an Online Initiative. Natl. Cancer Inst..

[9] Fournier D.M., Bazzell A.F., Dains J.E. (2018). Comparing Outcomes of Genetic Counseling Options in Breast and Ovarian Cancer: An Integrative Review. Oncol. Nurs. Forum..

[10] Ringwald J., Wochnowski C., Bosse K., Giel K.E., Schäffeler N., Zipfel S., Teufel M. (2019). Psychological Distress, Anxiety, and Depression of Cancer-Affected BRCA1/2 Mutation Carriers: A Systematic Review. Curr. Oncol. Rep..

[11] Aginnitopoulos K., Pepe G., Tsaousis G.N., Potska K., Bouzarelou D., Katseli A., Ntogka C., Meintani A., Tsoulos N., Giassas S. (2023). Copy Number Variations (CNVs) account for 10.8% of pathogenic variants in patients referred for hereditary cancer testing. Cancer Genom. Proteom..

[12] Litton J.K., Burstein H.J., Turner N.C. (2019). Molecular Testing in Breast Cancer. Am. Soc. Clin. Oncol. Educ. Book.

[13] Yamauchi H., Takei J. (2018). Management of hereditary breast and ovarian cancer. Int. J. Clin. Oncol..

[14] Frey M.K., Ahsan M.D., Badiner N., Lin J., Narayan P., Nitecki R., Rauh-Hain J.A., Moss H., Fowlkes R.K., Thomas C. (2022). What happens in the long term: Uptake of cancer surveillance and prevention strategies among at-risk relatives with pathogenic variants detected via cascade testing. Cancer.

[15] Bednar E.M., Sun C.C., Sheryl McCurdy S., Vernon S.W. (2020). Assessing relatives’ readiness for hereditary cancer cascade genetic testing. Genet. Med..

[16] Evans D.G.R., Binchy A., Shenton A., Hopwood P., Craufurd D. (2009). Comparison of proactive and usual approaches to offering predictive testing for BRCA1/2 mutations in unaffected relatives. Clin. Genet..

[17] Whitaker K.D., Obeid E., Daly M.B., Hall M.J. (2021). Cascade Genetic Testing for Hereditary Cancer Risk: An Underutilized Tool for Cancer Prevention. JCO Precis. Oncol..

[18] Kivimäki M., Batty G.D., Pentti J., Shipley M.J., Sipilä P.N., Nyberg S.T., Suominen S.B., Oksanen T., Stenholm S., Virtanen M. (2020). Association between socioeconomic status and the development of mental and physical health conditions in adulthood: A multi-cohort study. Lancet Public Health.

[19] Sarki M., Ming C., Aceti M., Fink G., Aissaoui S., Bürki N., Graffeo R., Heinimann K., Caiata Zufferey M., Monnerat C. (2022). The Cascade Consortium. Relatives from Hereditary Breast and Ovarian Cancer and Lynch Syndrome Families Forgoing Genetic Testing: Findings from the Swiss CASCADE Cohort. J. Pers. Med..

[20] Garcia C., Sullivan M.W., Lothamer H., Harrison K.M., Chatfield L., Thomas M.H., Modesitt S.C. (2020). Mechanisms to increase cascade testing in hereditary breast and ovarian cancer: Impact of introducing standardized communication aids into genetic counseling. J. Obstet. Gynaecol. Res..

[21] Lee K.H. (2023). Prediction of carrying a BRCA1 or BRCA2 mutation. Ann. Transl. Med..

[22] Yoshimura A., Imoto I., Iwata H. (2022). Functions of Breast Cancer Predisposition Genes: Implications for Clinical Management. Int. J. Mol. Sci..

[23] Seven M., Shah L.L., Yazici H., Daack-Hirsch S. (2022). From Probands to Relatives: Communication of Genetic Risk for Hereditary Breast-Ovarian Cancer and Its Influence on Subsequent Testing. Cancer Nurs..

[24] Heald B., Pirzadeh-Miller S., Ellsworth R.E., Nielsen S.M., Russell E.M., Beitsch P., Esplin E.D., Nussbaum R.L., Pineda-Alvarez D.E., Kurian A.W. (2023). Cascade testing for hereditary cancer: Comprehensive multigene panels identify unexpected actionable findings in relatives. Natl. Cancer Inst..

