# Supplementary material for: Only 32.3% of Breast Cancer Families with Pathogenic Variants in Cancer Genes Utilized Cascade Genetic Testing
Source: Cancers (Basel). 2023 Oct 30;15(21):5218. doi: 10.3390/cancers15215218 (PMC10649031; doi:10.3390/cancers15215218)
Supplement: Supplementary file 1 [file cancers-15-05218-s001.zip › cancers-2647019-supplementary.pdf]

Table S1. 52-genes included in this study.

**Gene**	**Reference sequence**
APC	NM_000038
ATM	NM_000051
ATR	NM_001184
AXIN2	NM_004655
BAP1	NM_004656
BARD1	NM_000465
BLM	NM_000057
BMPR1A	NM_004329
*BRCA1**	NM_007294
*BRCA2**	NM_000059
*BRIP1 **	NM_032043
CDH1	NM_004360
CDK4	NM_000075
CDKN2A (p14ARF, p16INK4a)	NM_000077, NM_058195
*CHEK2**	NM_007194
*EPCAM**	NM_002354
FAM175A	NM_139076
FANCA	NM_000135
FANCL	NM_001114636
FANCM	NM_020937
GALNT12	NM_024642
GEN1	NM_001130009
HOXB13:c.251G>A p.(G84E)	NM_006361
MEN1	NM_000244
MITF	NM_001354604
*MLH1**	NM_000249
MRE11	NM_005591
*MSH2**	NM_000251
MSH3	NM_002439
*MSH6**	NM_000179
*MUTYH**	NM_001128425
NBN	NM_002485
NF1	NM_000267
NTHL1	NM_002528
*PALB2**	NM_024675
PMS2	NM_000535
POLD1 (Exons 8-13)	NM_001256849
POLE (Exons 1-14)	NM_006231
PPP2R2A	NM_002717
PTEN	NM_000314
*RAD50**	NM_005732
RAD51B	NM_133509
*RAD51C**	NM_058216
*RAD51D**	NM_002878
RET	NM_020975
RNF43	NM_017763
RPS20	NM_001023
SMAD4	NM_005359
SMARCA4	NM_001128849
STK11	NM_000455
*TP53**	NM_000546
VHL	NM_000551

*Copy number variation analysis was performed on the following genes:

BRCA1, BRCA2, CHEK2, EPCAM (Exons 8, 9), MLH1, MSH2, MSH6, MUTYH, PALB2, RAD50 (Exons 1, 2, 4, 10, 14, 21, 23 and 25), RAD51C, RAD51D, and TP53.

Table S2. Breast cancer associated genes and risk category.

Gene	Risk category (Absolute risk)	Risk evidence
ATM	Moderate (20%–30%)	NCCN Guidelines®, Genetic/Familial High-Risk Assessment: Breast, Ovarian, and Pancreatic, Version 1.2024
BARD1	Moderate (17%–30%)	NCCN Guidelines®, Genetic/Familial High-Risk Assessment: Breast, Ovarian, and Pancreatic, Version 1.2024
BLM	Unspecified	PMID: 31614901, 20301572
BRCA1	High (>60%)	NCCN Guidelines®, Genetic/Familial High-Risk Assessment: Breast, Ovarian, and Pancreatic, Version 1.2024
BRCA2	High (>60%)	NCCN Guidelines®, Genetic/Familial High-Risk Assessment: Breast, Ovarian, and Pancreatic, Version 1.2024
BRIP1	Unspecified	NCCN Guidelines®, Genetic/Familial High-Risk Assessment: Breast, Ovarian, and Pancreatic, Version 1.2024
CDH1	High (41%–60%)	NCCN Guidelines®, Genetic/Familial High-Risk Assessment: Breast, Ovarian, and Pancreatic, Version 1.2024
CHEK2	Moderate (20%–40%)	NCCN Guidelines®, Genetic/Familial High-Risk Assessment: Breast, Ovarian, and Pancreatic, Version 1.2024
EPCAM	Low (<15%)	NCCN Guidelines®, Genetic/Familial High-Risk Assessment: Breast, Ovarian, and Pancreatic, Version 1.2024
FAM175A	Uncertain	PMID: 35353237, 22357538
FANCA	Uncertain	PMID:32235514
FANCL	Uncertain	PMID:32235514
FANCM	Uncertain	PMID:32235514
MLH1	Low (<15%)	NCCN Guidelines®, Genetic/Familial High-Risk Assessment: Breast, Ovarian, and Pancreatic, Version 1.2024
MRE11	Unspecified	PMID: 23586058
MSH2	Low (<15%)	NCCN Guidelines®, Genetic/Familial High-Risk Assessment: Breast, Ovarian, and Pancreatic, Version 1.2024
MSH6	Low (<15%)	NCCN Guidelines®, Genetic/Familial High-Risk Assessment: Breast, Ovarian, and Pancreatic, Version 1.2024
NBN	Unspecified	PMID: 33471991
NF1	Moderate (20%–40%)	NCCN Guidelines®, Genetic/Familial High-Risk Assessment: Breast, Ovarian, and Pancreatic, Version 1.2024
NTHL1	Unspecified	NCCN Guidelines®, Genetic/Familial High-Risk Assessment: Colorectal, Version 1.2023
PALB2	High (41%–60%)	NCCN Guidelines®, Genetic/Familial High-Risk Assessment: Breast, Ovarian, and Pancreatic, Version 1.2024
PMS2	Low (<15%)	NCCN Guidelines®, Genetic/Familial High-Risk Assessment: Breast, Ovarian, and Pancreatic, Version 1.2024
PTEN	High (40%–60%)	NCCN Guidelines®, Genetic/Familial High-Risk Assessment: Breast, Ovarian, and Pancreatic, Version 1.2024
RAD50	Unspecified	PMID: 21356067
RAD51C	Moderate (17%–30%)	NCCN Guidelines®, Genetic/Familial High-Risk Assessment: Breast, Ovarian, and Pancreatic, Version 1.2024
RAD51D	Moderate (17%–30%)	NCCN Guidelines®, Genetic/Familial High-Risk Assessment: Breast, Ovarian, and Pancreatic, Version 1.2024
SMARCA4	Unspecified	PMID: 21356067
STK11	High (32%–54%)	NCCN Guidelines®, Genetic/Familial High-Risk Assessment: Breast, Ovarian, and Pancreatic, Version 1.2024
TP53	High (>60%)	NCCN Guidelines®, Genetic/Familial High-Risk Assessment: Breast, Ovarian, and Pancreatic, Version 1.2024
